# Evaluation of Cholera Toxin B Subunit as a Novel Carrier Protein for Polysaccharide Conjugate Vaccines

**DOI:** 10.3390/vaccines13111159

**Published:** 2025-11-13

**Authors:** Chathuranga Siriwardhana, Aakriti Bajracharya, Florence Seal, Anup Datta, Subhash Kapre

**Affiliations:** 1 Bacterial Research and Development, Inventprise, Inc., Redmond, WA 98052, USA; 2 Immunology, Manufacturing Sciences and Technology, Inventprise, Inc., Redmond, WA 98052, USA; 3 Inventprise, Inc., Redmond, WA 98052, USA

**Keywords:** cholera toxin B subunit (CTB), polysaccharide–protein conjugates, carrier protein, CDAP conjugation, immunogenicity

## Abstract

**Background:** The immunogenicity of polysaccharide conjugate vaccines is critically influenced by the choice of carrier protein, which promotes a T-cell-dependent immune response mechanism leading to strong antibody production. In this study, the cholera toxin B subunit (CTB), a non-toxic pentameric protein, was evaluated as a novel carrier protein for pneumococcal polysaccharide antigens. **Methods**: Recombinant CTB was produced in *Escherichia coli* and purified using scalable chromatographic methods. Pneumococcal polysaccharides from serotypes 7F, 22F, and 33F were chemically activated with CDAP and conjugated to CTB. **Results:** The resulting glycoconjugates were characterized by SEC-MALS, confirming successful conjugation, high molecular weights, consistent polysaccharide-to-protein ratios, and acceptable endotoxin levels. Immunogenicity was assessed in rabbits following immunization with alum-adjuvanted formulations. **Conclusions**: Robust IgG responses were elicited by all CTB-based conjugates, with antibody levels found to be comparable to those induced by CRM197 conjugates, demonstrating the potential of CTB as a promising alternative for the next generation of conjugate vaccines.

## 1. Introduction

Vaccination continues to be one of the most impactful strategies for controlling and preventing infectious diseases worldwide. Through the widespread use of vaccines, diseases such as polio, measles, and tetanus have seen dramatic reductions in incidence, and in some cases, near-eradication [1,2,3]. Despite these successes, a significant challenge persists in the development of vaccines against pathogens with polysaccharide-rich outer capsules, such as Streptococcus pneumoniae. The pneumococcus is responsible for a range of serious illnesses, including pneumonia, meningitis, and septicemia, particularly affecting young children, the elderly, and immuno-compromised individuals [4,5,6]. While pneumococcal conjugate vaccines (PCVs) have significantly reduced the burden of pneumococcal disease, several limitations persist, including limited serotype coverage and the potential for carrier-induced epitopic suppression due to repeated use of the same carrier proteins. These challenges highlight the need for novel strategies in vaccine design, particularly the identification of alternative carrier proteins for conjugates vaccines [7,8,9,10].

Polysaccharide antigens by themselves elicit a T-cell-independent immune response, which typically results in short-lived antibody production and poor immunological memory [11]. This response is often inadequate in infants and older adults, the very population most vulnerable to invasive bacterial infections [11,12]. Conjugate vaccines were developed to overcome this limitation by covalently linking polysaccharide antigens to carrier proteins capable of eliciting a T-cell-dependent response. This strategy transforms the immune profile of the polysaccharide antigen, enabling the activation of helper T-cells, the generation of high-affinity antibodies, and the establishment of long-term immunological memory [13,14] While T-cell involvement underlies this mechanism, the resulting antibody responses serve as a key measurable indicator of vaccine effectiveness. The success of conjugate vaccines in reducing the prevalence of diseases caused by encapsulated bacteria, such as *Haemophilus influenzae* type b, *Neisseria meningitidis*, and *Streptococcus pneumoniae*, has validated this approach as a cornerstone of modern vaccinology [15,16,17].

Currently, the most commonly used carrier proteins in licensed conjugate vaccines include tetanus toxoid (TT), diphtheria toxoid (DT), and the non-toxic mutant CRM197 [18,19,20,21]. While these carriers are effective, their repeated use in multiple vaccines raises concerns about potential immune interference, carrier-induced epitopic suppression, and limited flexibility in antigen design [22,23,24]. These concerns have spurred interest in the development of novel carrier proteins that not only overcome these limitations but also provide additional immunological benefits, such as enhanced immunogenicity and broader applicability across different routes of administration [13].

One such promising candidate is cholera toxin subunit B (CTB), a non-toxic component of the cholera toxin complex produced by *Vibrio cholerae*. CTB is a pentameric protein known for its high affinity to GM1 ganglioside receptors expressed on epithelial cells. This binding capability has positioned CTB as a potent mucosal adjuvant and delivery system in oral and intranasal vaccines [25,26,27,28]. However, CTB’s utility extends beyond mucosal immunization. Its abilities to interact with antigen-presenting cells, stimulate dendritic cell maturation, and facilitate the presentation of linked antigens make it an attractive carrier protein candidate, even for parenterally delivered vaccines [29,30,31,32].

In this study, we explore the use of CTB as a carrier protein for a conjugate vaccine incorporating pneumococcal capsular polysaccharides. Importantly, rather than leveraging its mucosal adjuvanticity, we employed intramuscular administration to evaluate the systemic immune response elicited by CTB-conjugated polysaccharide antigens. This design allowed us to investigate CTB’s capacity to function effectively in a conventional vaccine delivery context and assess its potential advantages over traditional carrier proteins in stimulating a robust and protective immune response.

The rationale for selecting CTB in this context is twofold. First, CTB has been demonstrated in multiple preclinical studies to possess strong immunomodulatory effects, including the activation of Th1 and Th2 pathways and enhancement of antigen uptake by antigen-presenting cells [33,34,35,36]. These properties are desirable for a carrier protein, especially in conjugate vaccine platforms where the goal is to elevate a weak polysaccharide antigen to a fully immunogenic construct. Second, given the growing demand for new carrier proteins to avoid immune interference and enhance vaccine versatility, CTB offers a structurally and functionally distinct alternative to currently approved carriers [32,33,34].

While the majority of CTB research has focused on its application in mucosal vaccines targeting pathogens like *Helicobacter pylori* and enterotoxigenic *Escherichia coli*, relatively little is known about its performance in parenteral vaccines targeting non-mucosal pathogens [25,37,38,39]. Our work aims to fill this gap by assessing the immunogenicity of CTB-conjugated pneumococcal polysaccharides delivered via intramuscular injection.

The development of new pneumococcal vaccines remains a global priority, particularly in the face of rising antimicrobial resistance and serotype replacement following widespread use of current PCVs [40,41,42]. By evaluating CTB as a systemic carrier protein, this study seeks to expand the toolkit available for next-generation conjugate vaccine design. If proven effective, CTB could offer a path toward safer, more immunogenic, and more broadly applicable conjugate vaccines not only for pneumococcus but also for a wider array of polysaccharide-encapsulated bacterial pathogens [43,44,45,46,47,48].

In this study, we explore the conjugation strategy, characterize the immunological response, and compare its efficacy to that of a traditional carrier, CRM197. Importantly, we have also developed a robust method for large-scale cost-effective expression and purification of CTB, enabling its practical application in scalable vaccine manufacturing [48,49,50]. By exploring this innovative approach, we aim to contribute to the ongoing efforts in enhancing vaccine efficacy, reducing disease burden, and improving global access to life-saving immunizations.

## 2. Materials and Methods

### 2.1. Construction of Expression Plasmid

The gene sequence encoding the cholera toxin B subunit (CTB) was obtained from GenBank (ACCESSION EU828587 VERSION EU828587.1). An outer membrane protein A, OmpA (amino acid sequence: MKKTAIAIAVALAGFATVAQA) signal sequence was fused to the N-terminus of the CTB gene to direct periplasmic localization. The fusion construct was codon-optimized for expression in *Escherichia coli (E. coli)* using the Genscript codon optimization algorithm. The optimized gene was synthesized and cloned into the pET-28a(+) vector under the control of the T7 promoter by GenScript USA Inc. (Piscataway, NJ, USA). The resulting recombinant plasmid, designated OmpA-CTB-pET28a(+), was transformed into *E. coli* BL21(DE3) (New England Biolabs, Ipswich, MA, USA) cells for protein expression.

### 2.2. Protein Expression and Purification

A 1 mL vial from the master cell bank was used to inoculate 150 mL of Terrific Broth (Technova, Holister, CA, USA) containing 50 µg/mL kanamycin (Sigma-Aldrich, St. Louis, MO, USA). The culture was incubated at 37 °C until the optical density at 600 nm (OD_600_) reached 0.3, and the entire volume was transferred to a 10 L Eppendorf bioreactor containing 7 L of TB. The dissolved oxygen was maintained at 40%, and the pH was regulated at 7.0 using 30% ammonium hydroxide. Agitation was set at 250 rpm. A feed containing 50% glycerol and 60 g/L yeast extract was supplied continuously. Protein expression was induced with 1 mM Isopropyl β-D-thiogalactopyranoside (IPTG) (Technova, Holister, CA, USA), and fermentation proceeded for 18 h at 25 °C. Cells were harvested by centrifugation at 10,500× *g* for 1 h at 4 °C. Both the cell pellet and the supernatant were retained for further processing.

The cell pellet was resuspended in lysis buffer comprising 20 mM Tris and 2 mM EDTA at pH 6.0, supplemented with a protease inhibitor cocktail (Thermo Fisher Scientific, Waltham, MA, USA). The suspension was homogenized at 16,000–18,000 psi for two cycles while maintaining the temperature at 4 °C. The lysate was clarified by centrifugation at 10,500× *g* for 30 min at 4 °C, followed by filtration through a depth filter of 0.8 µM pore size.

Two purification schemes were evaluated. In scheme 1, the clarified lysate was subjected to immobilized metal affinity chromatography using a Ni^2+^ Sepharose resin (Cytiva, Marlborough, MA, USA). The column was equilibrated with 20 mM Tris and 150 mM NaCl at pH 7.4. The bound protein was washed with equilibration buffer containing 50 mM imidazole and eluted with 400 mM imidazole. In scheme 2, the lysate was first applied to a Tosoh sulfate 650 fast flow (Tosoh Corporation, Tokyo, Japan) column equilibrated with 20 mM Tris and 2 mM EDTA at pH 6.0. After washing with buffer containing 50 mM NaCl, protein was eluted using 500 mM NaCl. The eluate was further purified by Ni^2+^ affinity chromatography as described above. The supernatant fraction was processed only by Scheme 2. The protein was buffer exchanged using tangential flow filtration into 10 mM PB and 10% sucrose pH 7.0 and was stored at −80 °C until further use.

Protein identity and purity were confirmed by liquid chromatography–mass spectrometry (LC-MS) SDS PAGE (Native and denatured) and Western blotting. For intact mass analysis, CTB was separated on a protein bridged ethylene hybrid (BEH) size-exclusion chromatography (SEC) column (Waters Corporation, Milford, MA, USA) using ammonium acetate as the mobile phase and analyzed on a Waters ACQUITY time-of-flight (TOF) mass spectrometer. For peptide mapping, CTB was denatured, reduced, alkylated with iodoacetamide (Sigma-Aldrich, St. Louis, MO, USA), and digested with sequencing-grade trypsin (Waters Corporation, Milford, MA, USA). Peptides were separated by reverse-phase chromatography with a gradient of water and acetonitrile containing 0.1% formic acid on a C18 column (Waters Corporation, Milford, MA, USA) and analyzed by LC-MS.

### 2.3. Polysaccharide Fermentation and Purification

*Streptococcus pneumoniae* strains corresponding to serotypes 22F, 7F, and 33F were cultured in proprietary media at 36.5 ± 2.0 °C, with pH maintained at 7.30 ± 0.20 using sodium hydroxide and sodium carbonate. Cultures were harvested upon OD_600_ decline following treatment with 0.5% sodium deoxycholate. Crude lysates were treated sequentially with Denarase (c-Lecta, Leipzig, Germany), Lysobac (InVitria, Inc., KA, USA), and Proteinase K (Roche Diagnostics, Mannheim, Germany) at 36.5 °C to degrade nucleic acids and proteins. Clarified supernatants were purified by tangential flow filtration followed by hydrophobic interaction chromatography using Sartobind phenyl membrane (Sartorious, Gottingen, Germany), and we allowed a buffer exchange step. The purified polysaccharides were sterile filtered (0.22 µm) and stored at or below −60 °C. Prior to conjugation, molecular weights were adjusted by high-pressure homogenization to approximately 200 kDa for 22F, 145 kDa for 7F, and 211 kDa for 33F. Polysaccharides were solvent exchanged into water and characterized by nuclear magnetic resonance (NMR).

### 2.4. Conjugation, Purification, and Vaccine Formulation

Polysaccharide activation was performed using 1-cyano-4-dimethylaminopyridinium tetrafluoroborate (CDAP) (Sigma-Aldrich, St. Louis, MO, USA) following the method of Lees et al. [51]. Cyanation was carried out at a 1:1 polysaccharide-to-CDAP weight ratio in 0.25 M HEPES and 1 M NaCl at pH 9.0 for 7 to 10 min. CTB was then added at a 1:1 weight ratio, and the conjugation reaction was incubated overnight at room temperature. The reaction was quenched with glycine, and the conjugate was buffer exchanged into 10 mM phosphate buffer at pH 7.0.

Conjugates were analyzed for molecular size by SEC coupled with multi-angle light scattering (MALS). Polysaccharide concentrations were determined using the anthrone assay [52], and protein content was quantified using the Lowry (Pierce™ Modified Lowry Protein Assay Kit, Fischer Scientific, Waltham, MA, USA) method. Free polysaccharide content was measured by deoxycholate precipitation [53] followed by anthrone analysis. Endotoxin levels were measured using the Limulus amebocyte lysate (LAL) assay kinetic chromogenic method using the Endosafe^®^ Nexgen PTS™ system (Charles River Laboratories, Wilmington, MA, USA). Samples are mixed with LAL reagent and a chromogenic substrate. The resulting color intensity defines the endotoxin concentration when compared against the built-in standard curve.

Monovalent conjugates were combined to prepare a trivalent formulation containing each conjugate at 0.88 µg/mL based on polysaccharide concentration. The vaccine was formulated with 250 µg/mL aluminum phosphate, 10 mM phosphate buffer, 150 mM NaCl, and 0.02% Tween 80 (*v*/*v*).

### 2.5. Rabbit Immunization Study

Twelve- to fourteen-week-old New Zealand White rabbits (*n* = 5 per test group) were used for immunization studies conducted at Cocalico Biologicals, Inc, PA, USA (USDA Research License Number: 23-R-0089). On Day 0, each rabbit received a pre-bleed via venipuncture followed by intramuscular immunization with 0.5 mL of the formulated vaccine (0.44 µg of each conjugate). A test bleed was collected and a booster dose (0.5 mL) administered intramuscularly on Day 14. Animals were monitored daily for general health and adverse effects. On Day 28, rabbits were humanely euthanized and terminal bleeds collected for downstream immunological analysis.

### 2.6. IgG Quantification by Multiplex Bead-Based Immunoassay

Serum IgG antibodies specific to pneumococcal polysaccharide serotypes were quantified using a multiplexed bead-based immunoassay (Bio-Rad, Hercules, CA, USA). Immune sera collected from rabbits were analyzed for IgG concentrations against each of the three vaccine-included serotypes using Luminex technology. Quantification was performed with reference to the International Standard Serum 007sp (Lot #007sp007sp; NIBSC, UK).

All serum samples, including standards and controls, were centrifuged at 14,000–14,800 rpm for 5–10 min at 2–8 °C to remove particulates. Samples were diluted in Luminex assay buffer consisting of 0.2% bovine serum albumin (BSA), 0.05% sodium azide, and 0.1% Tween 20 in phosphate-buffered saline (PBS, pH 7.4)

Magnetic beads conjugated with the corresponding pneumococcal polysaccharides were diluted 1:500 in Luminex buffer, and 50 µL of this bead suspension was added per well to a pre-wetted filter plate. Diluted serum samples were then transferred to the wells containing beads. The plates were incubated at 37 ± 3 °C for 60–80 min with continuous shaking at 100 ± 20 rpm. Following incubation, the plates were washed to remove unbound antibodies, and 50 µL of a phycoerythrin-conjugated, species-specific secondary antibody diluted 1:250 was added to each well. Plates were further incubated for 30–40 min under the same temperature and shaking conditions.

After a final series of washes, 100 µL of Luminex buffer was added to each well, and the plates were analyzed using the Bio-Plex 200 system (Bio-Rad, Hercules, CA, USA). Data acquisition and analysis were performed using Bio-Plex Manager software version 6.2, and IgG concentrations were interpolated from standard curves generated with the reference serum.

## 3. Results

### 3.1. E. coli Fermentation and Protein Purification

*E. coli* cells harboring the plasmid OmpA-CTB-pET were cultivated to a final OD_600_ of 90. This high-density cultivation yielded a weT-cell mass of approximately 250 g/L, reflecting robusT-cell growth and protein production. Post-harvest, approximately 9 L of supernatant was collected for downstream processing.

**Figure 1:**
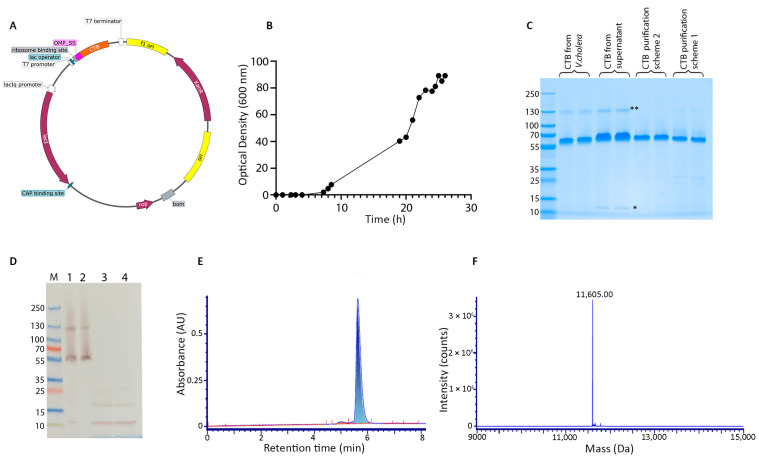
Expression, purification, and characterization of recombinant CTB. (**A**) Schematic representation of the expression plasmid encoding cholera toxin B subunit (CTB) under control of the T7 promoter, with an OmpA signal sequence for periplasmic export. (**B**) Growth curve of the recombinant E. coli strain showing optical density increase over time, indicative of successful cell growth. (**C**) Native SDS-PAGE analysis of CTB expression and purification. Lanes show CTB from V. cholerae (reference), CTB from recombinant culture supernatant, and CTB purified using two different chromatographic schemes (* and ** represent monomers and multimers, respectively). (**D**) Western blot analysis using anti-CTB antibodies confirming the identity of purified CTB in various fractions. Lane M: molecular weight marker; Lanes 1: non-denatured CTB from *V. cholera*, 2: native CTB from purification scheme 2, Lane 3: denatured CTB from *V. cholera*, Lane 4: denatured CTB from purification scheme 2. (**E**) Analytical HPLC SEC showing a single, symmetric peak corresponding to CTB. (**F**) Mass spectrometry analysis indicating a dominant peak at 11,605 Da, consistent with the expected monomeric mass of CTB.

Two chromatographic purification schemes were evaluated. In the first purification scheme, the lysate was subjected to a single-step immobilized Ni^2+^ affinity chromatography procedure. This approach yielded approximately 3.7 g of purified protein per kilogram of weT-cell mass. Analysis of the purified protein by native SDS-PAGE revealed a product purity of 92%. The protein was observed to assemble into a stable pentameric quaternary structure, with a total molecular weight of approximately 55 kDa (Figure 1C). Under denaturing conditions, the protein migrated as a single band corresponding to a molecular weight of approximately 11 kDa, consistent with its monomeric form (Supplementary Figure S1). This pentameric and monomeric molecular weight was comparable to that of CTB derived from *V. cholerae*, confirming the expected size of the monomeric unit. To further enhance purity, a second purification scheme was employed, which incorporated an additional cation exchange chromatography step prior to Ni^2+^ affinity chromatography. This modification resulted in a significant improvement in product purity, achieving >99% purity as determined by native SDS-PAGE. The yield from this two-step process was approximately 3.6 g of purified protein per kilogram of wet cell mass, comparable to the yield obtained from Scheme 1. Both purification schemes successfully isolated CTB, but Scheme 2, which included the additional cation exchange chromatography step, provided a significant advantage in terms of product purity (>99%) while maintaining a high yield.

The supernatant collected after cell harvest was also purified using process utilized in scheme 2. While the purity of the protein isolated from the supernatant was similarly high, the yield was notably lower, at about 0.2 g of purified protein per liter of supernatant. Analysis of the supernatant-derived protein revealed a minimal presence of monomeric and multimeric forms in addition to the pentameric form (Figure 1C), suggesting potential differences in protein stability when secreted to the media during bacterial growth in a manufacturing scenario.

The protein isolated from the lysate through Scheme 2 was further characterized using Western blotting and liquid chromatography–mass spectrometry (LC-MS). Western blot analysis using an anti-cholera toxin antibody (Abcam, Waltham, MA, USA) revealed a signal comparable to that of the CTB from *V. cholerae*, confirming the presence of a similar epitope. LC-MS analysis revealed an intact mass of 11.6 kDa for the protein (Figure 1F). To confirm the identity of the protein, it was subjected to trypsin digestion followed by peptide mapping. This analysis achieved 100% sequence coverage, unequivocally confirming the identity of the protein as CTB and validating the accuracy of the expression and purification process (Supplementary Figure S2).

### 3.2. Conjugation of Pneumococcal Polysaccharide with CTB

**Figure 2:**
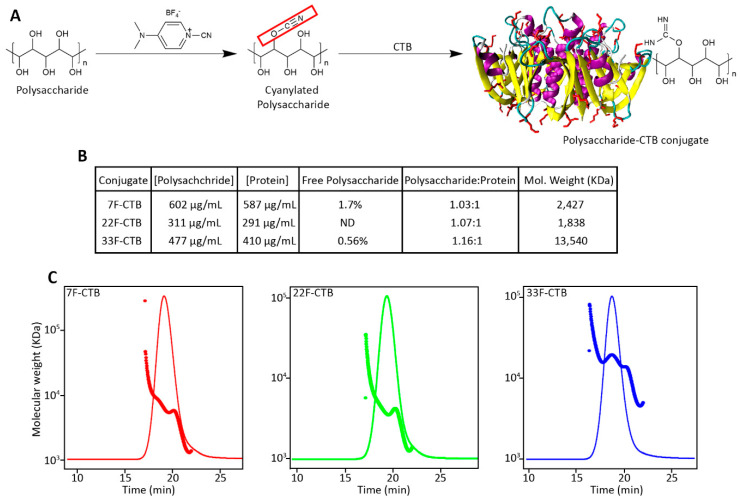
Characterization of polysaccharide-CTB conjugates synthesized. (**A**) Schematic representation of the conjugation strategy. Polysaccharides were first activated by cyanylation using 1-cyano-4-dimethylaminopyridinium tetrafluoroborate (CDAP), followed by covalent coupling to the cholera toxin B subunit (CTB) to generate polysaccharide–CTB conjugates. (**B**) Quantitative analysis of conjugate composition. Polysaccharide and protein concentrations were measured post-conjugation. Free (unconjugated) polysaccharide levels were determined by precipitating protein. Molecular weights were estimated using SEC-MALS. (**C**) Size exclusion chromatography–multi-angle light scattering (SEC-MALS) profiles of 7F-CTB (red), 22F-CTB (green), and 33F-CTB (blue) conjugates, showing elution time vs. molecular weight. Each profile indicates successful conjugation with distinct size distributions and molecular masses for each serotype.

Three pneumococcal serotypes were used for this study, PNU 7F (Figure S3), PNU22F (Figure S4), and PNU33F (Figure S5). The polysaccharides derived from these serotypes were chemically conjugated to CTB using a cyanate ester-mediated coupling reaction. The purified conjugates were subjected to size-exclusion chromatography coupled with multi-angle light scattering (SEC-MALS) to assess their molecular mass distributions (Figure 2C). The average molar masses of the conjugates were determined to be 2427 kDa for 7F-CTB, 1838 kDa for 22F-CTB, and 13,540 kDa for 33F-CTB.

The total polysaccharide content in the conjugates was quantified using the anthrone assay, while the total protein content was determined using the Lowry protein assay. The polysaccharide-to-protein ratio (*w*/*w*) in the purified conjugates was approximately 1:1. To evaluate the presence of unconjugated polysaccharide, samples from each conjugate were treated with sodium deoxycholate followed by hydrochloric acid precipitation. The analysis revealed that the amount of free polysaccharide remaining after purification was less than 2% for all conjugates. Additionally, the endotoxin content of the purified conjugates, as measured by the LAL assay, was found to be below the acceptable threshold of 5 endotoxin units/mL. These results confirm the successful conjugation and purification of the CTB-polysaccharide conjugates, with minimal residual unconjugated polysaccharide and endotoxin contamination.

### 3.3. Pneumococcal Polysaccharide Conjugated to CTB Is Comparatively Immunogenic to CRM197 Conjugates

To evaluate the immunogenicity of CTB as a novel carrier protein in pneumococcal conjugate vaccines, rabbits (*n* = 5) were immunized with polysaccharide conjugates of serotypes 7F, 22F, and 33F using CTB as the carrier. The conjugates were alum-adjuvanted and formulated. Polysaccharides conjugated with CRM197 using same CDAP chemistry were used as a comparator. Animals received two doses, on Days 0 and 14, and IgG concentrations against each serotype were measured on Days 14 and 28 using a multiplex ELISA assay.

**Figure 3:**
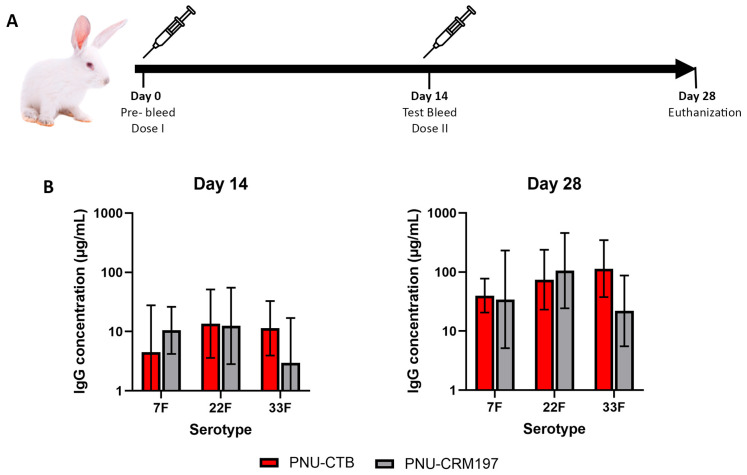
Immunogenicity of CTB and CRM197-conjugated pneumococcal vaccines in rabbits. (**A**) Schematic overview of the immunization protocol. Rabbits were immunized intramuscularly on Days 0 and 14 with alum-adjuvanted conjugate vaccines containing pneumococcal polysaccharides (7F, 22F, and 33F) coupled to either cholera toxin B subunit (CTB) or CRM197. (**B**) Serotype-specific IgG concentrations measured on Days 14 and 28 by multiplex bead-based immunoassay. Both CTB and CRM197-conjugated vaccines elicited measurable IgG responses following the prime and boost immunizations. Statistical comparisons were performed using unpaired two-tailed *t*-tests. A *p*-value of <0.05 was considered significant. No significant differences were observed between groups.

Following a single immunization, both CTB and CRM197-conjugated pneumococcal vaccines elicited measurable IgG responses against all three tested serotypes by Day 14 (Figure 3B). The IgG concentrations induced by the CTB conjugated formulations were generally comparable to those observed with the CRM197 conjugates across serotypes 7F, 22F, and 33F. No statistically significant differences were detected between the groups at this timepoint.

By Day 28, following administration of the booster dose, both vaccine groups exhibited a marked increase in serotype-specific IgG concentrations. The booster response was evident across all three serotypes for both CTB and CRM197-conjugated formulations. Both types of conjugates elicited robust post-boost responses, with antibody titers increasing consistently across serotypes (Figure 3B). Overall, the magnitude and kinetics of the booster response were comparable between the two carrier proteins, indicating that CTB supports effective immunological memory and recall upon secondary immunization.

## 4. Discussion

The current study demonstrates the feasibility and effectiveness of using CTB as an alternative carrier protein in pneumococcal conjugate vaccines, highlighting its potential to substitute the widely used CRM197. The research encompassed high-yield CTB expression in *E. coli*, development of scalable purification methods, successful conjugation with clinically relevant pneumococcal polysaccharides, and comparative immunogenicity evaluation in an animal model.

High-density fermentation of recombinant *E. coli* expressing CTB achieved excellent productivity, generating approximately 3.6–3.7 g of purified protein per kilogram of wet biomass. A unique feature of this process is that purification was accomplished through Ni^2+^ affinity chromatography without the addition of a polyhistidine tag. Instead, CTB’s native histidine residues located on its surface served as an intrinsic metal-binding site, enabling efficient capture on the Ni^2+^ matrix [54]. This property, rarely exploited in recombinant CTB workflows, eliminates the need for artificial tags and subsequent cleavage steps while preserving the structural integrity of both termini regions. Comparable tag-free metal affinity purification of CTB has only been described in limited biochemical reports [55]; the current work extends these findings by demonstrating scalability, reproducibility, and an exceptionally high purity (>99%) suitable for vaccine-grade material. The structural confirmation by LC-MS, peptide mapping, and SDS-PAGE verified that the recombinant protein was identical to the native *V. cholerae* CTB, consistent with earlier studies showing that recombinant CTB forms a correctly folded pentameric structure in *E. coli* [56,57]. The recovery of CTB from the culture supernatant, while successful, resulted in a substantially lower yield compared to periplasmic extraction. The reduced yield indicates that secretion efficiency is the main limiting factor of the extracellular expression system. Further optimization of secretion efficiency would therefore be required before adopting secretion-based expression systems for large-scale manufacturing.

Efficient conjugation of pneumococcal polysaccharides (7F, 22F, and 33F) to CTB was achieved using CDAP-mediated coupling chemistry. The resulting conjugates displayed high molecular weights (1.8–13.5 MDa) and balanced polysaccharide-to-protein ratios near 1:1 (*w*/*w*), with less than 2% unconjugated polysaccharide after purification. These characteristics align closely with established profiles of CRM197 or tetanus toxoid-based conjugates prepared with similar chemistries [48]. A comparable coupling efficiency has also been observed in previous studies utilizing CTB as a conjugation partner with dextran, *Haemophilus influenzae* type b, or Group B *Streptococcus* capsular polysaccharide [48,49,50], further validating CTB’s chemical compatibility and structural stability under conjugation conditions. The uniform SEC–MALS profiles observed in this study confirm that CTB tolerates activation and coupling without aggregation or degradation, an important prerequisite for consistent manufacturability.

The immunogenicity results demonstrated that CTB-based conjugates elicited strong serotype-specific IgG responses that were statistically indistinguishable from those induced by CRM197 conjugates. Both vaccine groups generated measurable antibody titers following a single immunization, and booster immunization on Day 14 led to marked secondary increases across all three serotypes. The absence of significant differences between CTB and CRM197 formulations suggests that CTB provides sufficient T-cell help to support polysaccharide-specific IgG production. Although cellular immune parameters were not directly evaluated here, the strong booster effect indicates successful conversion of the polysaccharide antigens from a T-cell-independent to a T-cell-dependent form.

Despite these promising results, several limitations should be acknowledged. The current immunogenicity assessment focused solely on total IgG concentrations as the measurable endpoint. Analyses of antibody avidity or affinity would provide additional insights into the quality of the antibody response. Similarly, anti-CTB antibody titers were not evaluated, which would help determine whether strong carrier-specific immunity develops and how it might influence booster responses or multivalent formulations. Cellular assays evaluating T-cell activation and cytokine secretion would further elucidate the mechanism by which CTB promotes antibody production. These evaluations, along with challenge models assessing protective efficacy, are planned for future studies to complement the present findings.

From a process and manufacturing standpoint, CTB offers tangible advantages. The recombinant expression system avoids detoxification and complex purification, and the use of native histidine-mediated affinity purification simplifies downstream processing and lowers the cost. This tag-free, high-yield system therefore provides a scalable route for producing a carrier protein suitable for low- and middle-income country vaccine programs where affordability and simplicity are essential. These attributes, coupled with CTB’s structural stability and demonstrated immunogenicity, position it as an attractive component for future conjugate vaccine platforms.

## Data Availability

The data is available from the corresponding author on request.

## Supplementary Materials

The following supporting information can be downloaded at: https://www.mdpi.com/article/10.3390/vaccines13111159/s1, Figure S1. Denaturing SDS-PAGE analysis of CTB expression and purification, Figure S2. Peptide mapping of purified CTB using triptic cleavage, representing 100% coverage. Figure S3. 1H NMR Spectra of homogenized pneumococcal polysaccharide serotype 7F. Figure S4. 1H NMR Spectra of homogenized pneumococcal polysaccharide serotype 22F. Figure S5. 1H NMR Spectra of homogenized pneumococcal polysaccharide serotype 33F.

## Abbreviations

The following abbreviations are used in this manuscript:

CTB	Cholera toxin subunit B
CDAP	1-Cyano-4-dimethylaminopyridinium tetrafluoroborate
CRM197	Cross-reacting material 197
OMPA	Outer membrane protein A
IPTG	Isopropyl β-D-1-thiogalactopyranoside
IgG	Immunoglobulin G
