# Supplementary material for: Evaluation of Cholera Toxin B Subunit as a Novel Carrier Protein for Polysaccharide Conjugate Vaccines
Source: Vaccines (Basel). 2025 Nov 13;13(11):1159. doi: 10.3390/vaccines13111159 (PMC12656476; doi:10.3390/vaccines13111159)
Supplement: Supplementary file 1 [file vaccines-13-01159-s001.zip › vaccines-3943045-supplementary.pdf]

Supplementary Figures

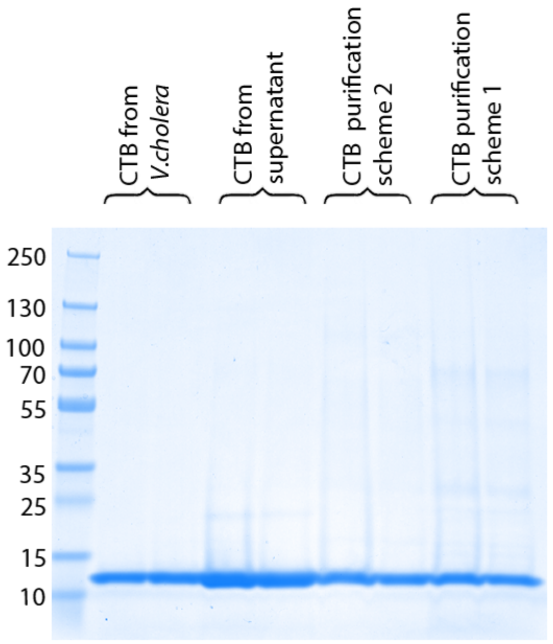

Supplementary Figure S1. Denaturing SDS-PAGE analysis of CTB expression and purification. Lanes show CTB from *V. cholerae* (reference), CTB from recombinant culture supernatant, and CTB purified using two different chromatographic schemes.

Item name: CTB_pep_map_alkylated 60 min, Sample position: 1:D,3, Replicate number: 1

.	Peptide	Fragment label	Modifiers	Observed mass (Da)	Mass error (ppm)	Observed RT (min)	MS Response	Peptide Match Score
1	TPQNITDLCAEYHNTQIHTLNDK	1:T1&	Carbamidomethyl C [9]	2726.2924	3.1	17.17	96389440	121.60
2	EMAIITFK	1:T4		952.5199	2.8	19.96	66381400	88.34
3	IFSYTESLAGK	1:T2		1215.6298	3.5	15.98	64175328	121.67
4	IAYLTEAK	1:T10		908.5118	3.3	11.34	58986096	74.12
5	NGATFQVEVPGSQHIDSQK	1:T5		2041.9994	2.8	16.35	54330052	111.21
6	LCVWNNK	1:T12&	Carbamidomethyl C [2]	933.4637	2.8	11.28	44403168	65.33
7	TPHAIAAISMAN	1:T13		1196.6128	3.0	15.52	43993752	85.87
8	NGATFQVEVPGSQHIDSQKK	1:T5-6		2170.0968	3.7	14.84	25816368	100.09
9	MKDTLR	1:T8-9		763.4154	3.1	2.49	23320456	86.39
10	AIER	1:T7		488.2837	2.0	0.92	4718571	24.26
11	DTLR	1:T9		504.2787	2.0	1.20	3630854	24.83
12	REMAIITFK	1:T3-4		1108.6214	2.8	16.77	703695	49.55
13	VEK	1:T11		375.2246	2.0	0.67	314998	23.55
14	IFSYTESLAGKR	1:T2-3		1371.7325	4.2	14.21	264816	59.65

CTB **Coverage: 100%**

```
1: 1 to 80     TPQNITD EYHNTQI NDKIFSY LAGKREM TFKNGAT EVPGSQH QKKAIER TLRIAYL
               LCA     HTL     TES     AII     FQV     IDS     MKD     TEA
1: 81 to 103   KVEKLCV KTPHAIA MAN
               WNN     AIS
```

Supplementary Figure S2. Peptide mapping of purified CTB using triptic cleavage, representing 100% coverage

7F 1H NMR

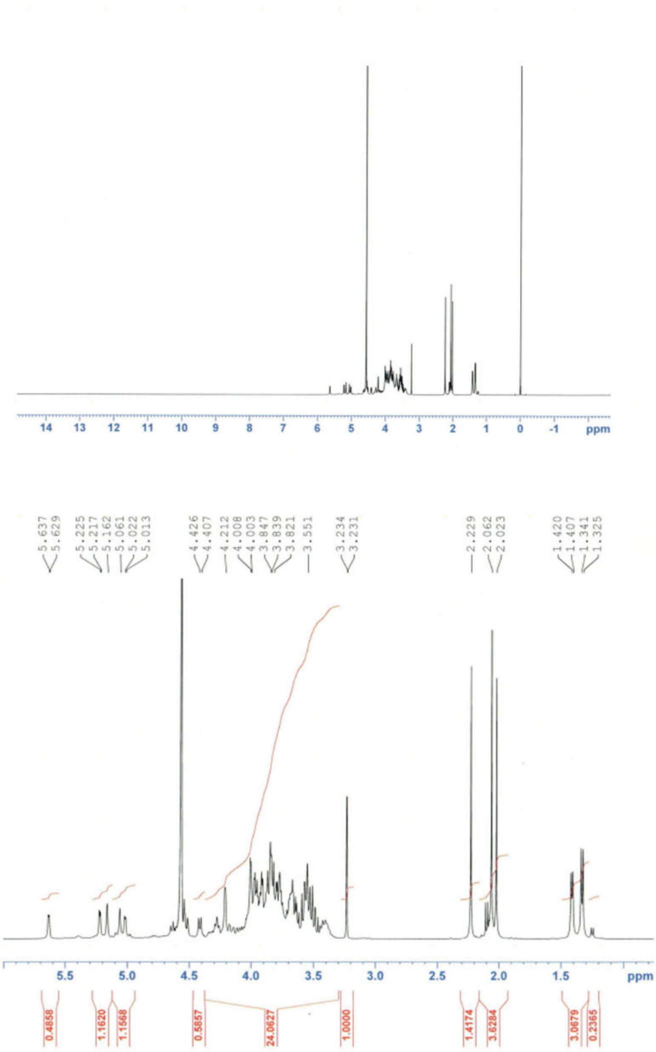

Supplementary Figure S3. 1H NMR Spectra of homogenized pneumococcal polysaccharide serotype 7F

22F 1H NMR

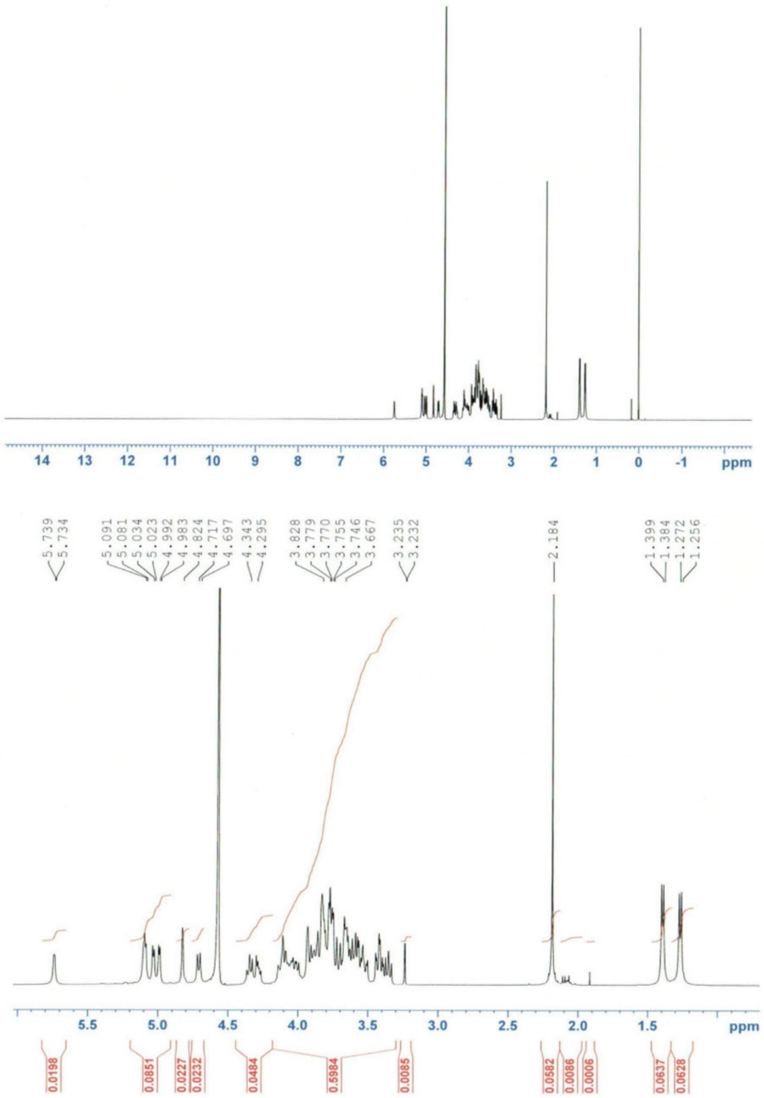

Supplementary Figure S4. 1H NMR Spectra of homogenized pneumococcal polysaccharide serotype 22F

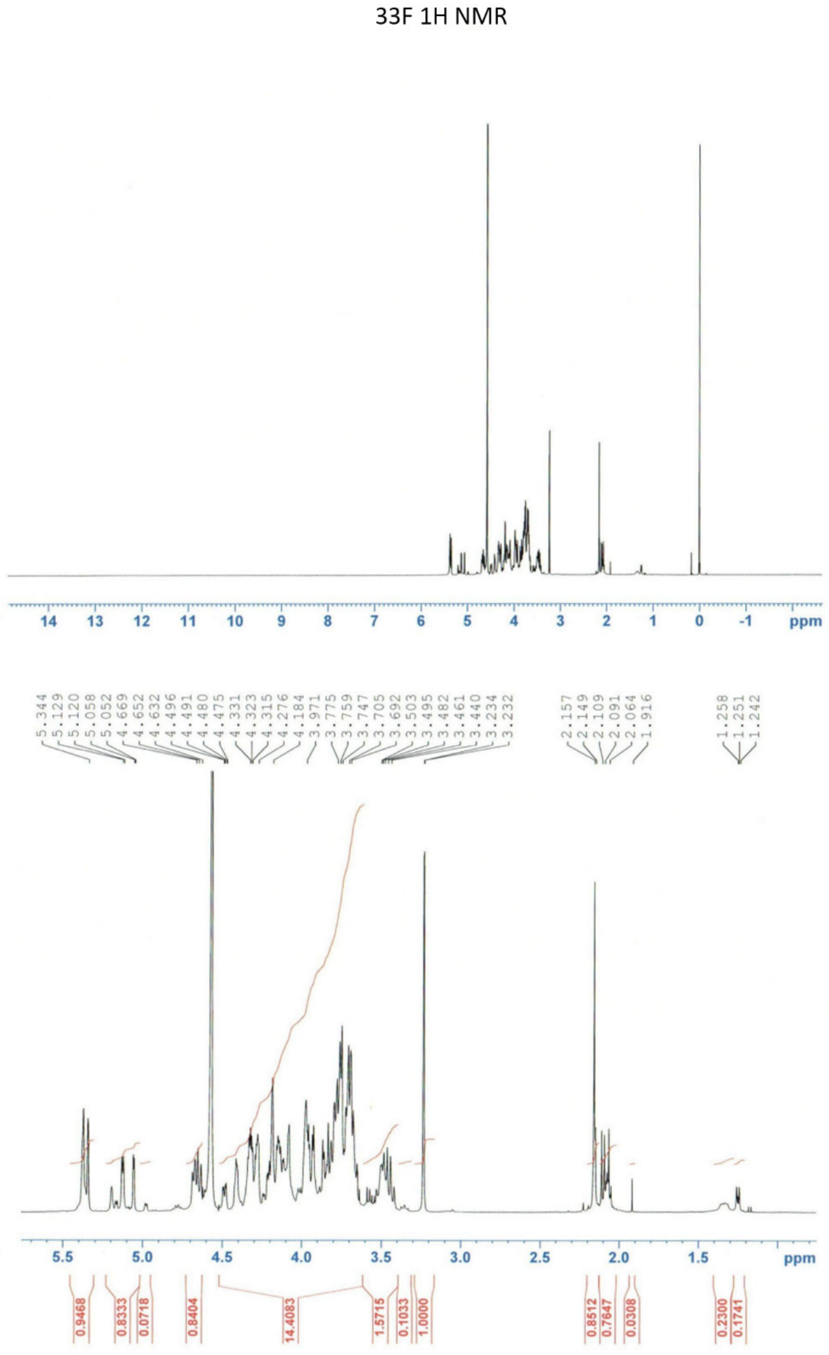

Supplementary Figure S5. 1H NMR Spectra of homogenized pneumococcal polysaccharide serotype 33F
